# Supplementary material for: Factors affecting postnatal care service utilization in Pyuthan district: A mixed method study
Source: PLoS One. 2024 Sep 6;19(9):e0307772. doi: 10.1371/journal.pone.0307772 (PMC11379213; doi:10.1371/journal.pone.0307772)
Supplement: S1 File — (DOCX) [file pone.0307772.s003.docx]

**Researcher's Reflexivity**

The researcher, a public health graduate familiar with the study site, has experience in building rapport with community members. A letter from School of Public Health, Patan Academy of Health Science helped obtaining approval from the municipality and rural municipality. Additionally, the approval letters from these municipalities eased the process of obtaining the list of study participants from health facilities. However, due to poor maintenance of the registers in the health facilities, preparing the sampling frame took longer than expected. Adverse weather conditions further complicated the fieldwork.

As the researcher was relatively inexperienced in qualitative research, initial interviews faced issues with ice-breaking and maintaining the flow of conversation. These issues were resolved after seeking advice from the guide and co-guide. Judgmental sampling was not consistently maintained, as health facility staff assisted in selecting some FCHVs for qualitative interviews. The researcher’s personal relationships with some health workers at the study site were not disclosed to any study subjects throughout the interviews.

Maintaining privacy was challenging in some cases, as the mothers-in-law or husbands of postnatal mothers attempted to listen to the conversations. Although requests were made to allow the mothers to speak privately, privacy could not be fully assured due to the virtual method of interviewing. Despite scheduling appointments at convenient times for the postnatal mothers, disturbances from their children occurred during some interviews. In such cases, interviews were either paused or rescheduled for another appropriate time. Network disturbances also occurred, necessitating repetition of questions if participants' voices were unclear. This may have caused participants to lose interest, potentially resulting in missed original information. Due to the busy schedules of the participants, some interviews were conducted at night.

Although some direct probing occurred during interviews, the researcher tried to remain as neutral as possible. All codes and code categories generated during qualitative data analysis were based on participants' statements, not on the researcher’s presumptions.
