# Supplementary material for: Factors affecting postnatal care service utilization in Pyuthan district: A mixed method study
Source: PLoS One. 2024 Sep 6;19(9):e0307772. doi: 10.1371/journal.pone.0307772 (PMC11379213; doi:10.1371/journal.pone.0307772)
Supplement: Attachment — Submitted filename: renamed_fac7f.docx [file pone.0307772.s004.docx]

**Factors affecting postnatal care service utilization in Pyuthan district: A mixed method study**

**Introduction**

1.I suggest that add the current situation of postnatal care globally in the second paragraph of the Introduction , rather than only describing the current situation in Nepal only.

2. There are few description in the status of postpartum care in Pyuthan.I suggest that describe the problems in the utilization of postnatal care services in Pyuthan through data.Then the purpose of this study is described.

**Materials and method**

1.Why do you choose Jhimruk Rural Municipality and Pyuthan Urban Municipality of Pyuthan district for research.

2.The interview outline of the qualitative survey or the main framework of the interview were not described.I suggest adding framework.

3.I suggest that the mixed method is specifically described, as it appears in the title and may be important.

**Results**

1. In the analysis of qualitative results, I suggest that attention paid to the analysis of the reason of certain phenomena, for example, 318 PNC home visits only described the current situation, before leaving the hospital, only the mother who gave birth in the health care institution received the first PNC examination. No follow-up tests were performed on the third and seventh days. So why wasn't there a follow-up examination? The interviews could have been semi-open. The purpose of finding the real reasons were and how to fix them. In line 326 Incentives, the same problem is suggested to be added.
